# A Feasible Method of Angiogenesis Assessment in Gastric Cancer Using 3D Microvessel Density

**DOI:** 10.1155/2018/7813729

**Published:** 2018-04-03

**Authors:** Lu Zhang, Fei Zheng, Zhigang Peng, Zijing Hu, Zhi Yang

**Affiliations:** ^1^School of Biomedical Engineering, Capital Medical University, Beijing, China; ^2^Beijing Key Laboratory of Fundamental Research on Biomechanics in Clinical Application, Capital Medical University, Beijing, China; ^3^Beijing Friendship Hospital, Capital Medical University, Beijing, China; ^4^Department of Pathology, School of Basic Medical Science, Capital Medical University, Beijing, China

## Abstract

**Background:**

Cancer stem cell (CSC) promotes angiogenesis which plays an important role in tumor occurrence, growth, and metastasis. Accurately, quantifying the tumor vasculature can help understanding CSC characteristics and improve cancer diagnosis, therapy planning, and evaluation. The objective of this study is to present a method for improved angiogenesis assessment.

**Methods:**

We proposed a three-dimensional microvessel density (3D MVD) to evaluate tumor angiogenesis and tested it in animal models. Six male Balb/c nude mice were divided into normal group and tumor group. The mice in tumor group were orthotopically implanted human gastric cancer, cell line BGC-823. The phase-contrast images were collected at Shanghai Synchrotron Radiation Facility BL13W beamline, which has much higher soft-tissue contrast and spatial resolution than conventional X-ray. After volume reconstruction and vessel extraction, the 3D models of the angiogenesis were established for MVD calculation.

**Results:**

The results showed that the proposed 3D MVD is positively correlated with the pathological changes of the microvessels. It took the advantage of high resolution of the phase-contrast imaging and added three-dimensional information to the existing MVD measure.

**Conclusions:**

Our study presents a feasible approach for better understanding of tumor angiogenesis. It may provide doctors and scientists a better tool for cancer investigation and improving medical outcomes.

## 1. Background

Gastric cancer is one of the most common malignancies in the world. Half of the cases occurred in Eastern Asia, especially in China. Till now the mortality rate of gastric cancer is still very high worldwide (8.8% of the total cancer death) [1], and the 5-year survival rate is only about 29% [2]. Early diagnosis and effective intervention are critical factors to improve the medical outcomes [3].

In recent studies, cancer stem cell (CSC) was identified in gastric cancer. It is believed that CSC is associated with neovascularization which nourishes tumor growth and becomes the major contributor to chemoresistance, tumor recurrence, and metastasis [4, 5]. A quantitative measure of angiogenesis can provide insight for CSC characteristics as well as play a pivotal role in cancer diagnosis, therapy planning, and evaluation. However, how to accurately quantify tumor vasculature still remains a challenging task.

Phase-contrast computed tomography (PC-CT) can produce three-dimensional (3D) microvascular information with high contrast and resolution. It detects the refraction information of the X-ray beam when crossing different materials. The information is much stronger than the conventional attenuation-based imaging and provides superior soft-tissue contrast [6]. Over the last decade, several studies have reported the applications of phase-contrast imaging (PCI) in microvascular imaging, such as depiction of brain microvasculature after ischemic injury, differentiation of breast cancer from benign one, and vascular indication of liver fibrosis [7–9]. Tang et al. reported that they found some microvessels and cancerous ulcer clearly in the gastric mucosa using PCI [10]. Although microvessels have been imaged with regard to some carcinomas, the spatial morphology of gastric cancer vasculature has not been investigated in detail.

On the other hand, for quantitatively assessing tumor angiogenesis, microvessel density (MVD) is considered to be a valuable parameter. A series of studies demonstrated that MVD is a strong indicator for tumor metastasis and consequently prognosis [11–13]. Nonetheless, there is no standard method to obtain MVD. Weidner et al. provided the MVD of breast cancer by immunohistochemistry (IHC) to measure the number of vessels in hotspots [14]. Other researchers evaluated MVD by altering the microscopic magnifications or IHC antibody (CD34, CD105, etc.) [15]. These methods have produced inconsistent results, which affect the reliability of MVD as the indicator of cancer prognosis. The MVDs were only from one or a few selected histological slices. The two-dimensional (2D) data may not be sufficient to demonstrate the complete structures of the tumor vasculature.

The purpose of this study was to use PCI to give a complete view of gastric cancer vasculature and propose a new 3D MVD parameter, which can demonstrate the microvessel distribution more comprehensively, and therefore provide a new way for the evaluation of gastric cancer therapy by imaging method. The details of the experiments and data processing are presented in the following section. In Results, we assess the value of 3D MVD in evaluating the angiogenesis of gastric cancer, then conclude the paper.

## 2. Methods

### 2.1. Animal Model Preparation

Seven adult male Balb/c nude mice weighing 15–25 g were used in this experiment. Six of them were randomly divided into normal group (*n* = 3) and tumor group (*n* = 3), and the other one was used for subcutaneous tumor implantation. During the experiment, all the mice were raised under SPF (Specific Pathogen Free) conditions, 12 h-day and night cycles, and free access to food and water. All experiments and procedures carried out on the animals were approved by the Animal Welfare Committee of Capital Medical University.

Tumor cell suspension (1 × 10^7^ cells/100 *μ*l) of human gastric cancer, cell line BGC-823, was subcutaneously injected into a male nude mouse (Balb/c). After the tumor diameter grew to 1-2 cm, we resected and cut it into fragments (1 mm^3^) for the next orthotopic implantation.

For the three mice in the tumor group, one tumor fragment for each mouse was orthotopically implanted onto the subserosa of the stomach with an 8-0 surgical suture. Then, we closed the abdominal wall and the skin. About one week later, the resulting orthotopic tumor diameter grew to 0.5 cm, which can be touched subcostal. Mice in the control group did not receive any operation.

It is known that PC-CT can provide better contrast to the soft tissues; however, we still applied contrast agent to further enhance the microvessels for robust MVD measurement. In our experiment, barium sulfate suspension was used because it can deposit in vessels for a relatively long period. Before imaging, all the mice were anesthetized by 10% chloral hydrate, 0.4 ml/100 g, i.p. Then, we opened their thoracic cavity to expose the heart, made an injection on the posterior end of the left ventricle by a 21-gauge blunt needle, which connected to a transfusion facility with physiological saline solution, and subsequently made an incision to the animal's right atrium. After about 5 min, when the lung and liver turned white, we removed the transfusion facility and changed to a 10 ml syringe with barium sulfate suspension (20% *v*/*v*), which was finely ground to enter the microvessels. Finally, the stomach samples were isolated and quickly preserved in formalin solution.

For the purpose of verifying the imaging results' accuracy, after imaging experiment, the samples were embedded into paraffin, cut into 5 *μ*m thick sections, and stained with hematoxylin and eosin (H&E).

### 2.2. Phase-Contrast Computed Tomography

**Figure 1:**
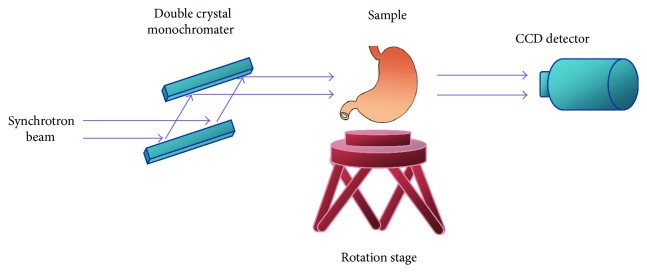
The schematic of imaging setup.

The phase-contrast images were collected at Shanghai Synchrotron Radiation Facility (SSRF) BL13W beamline. SSRF is one of the advanced third-generation light sources in the world. The imaging system in BL13W is relatively simple and has low optical device requirements. The schematic of imaging setup was shown in Figure 1. The synchrotron beam with high energy and collimation came from the storage ring first reached the double-crystal monochromator. The beam was therefore selected to a single energy ranging from 8 keV to 72.5 keV. The monochromatic beam then passed through the imaging sample which was positioned on six degrees of freedom motion platform. The sample can rotate 360° in the horizontal plane to make a CT scan. The transmitted beam carried the information of the sample traveled for a specific distance. Within this distance, Fresnel diffraction happened and therefore lead to an improved image contrast in different tissues. Finally, the beam was received and transformed into visible light by a CCD detector. In our experiment, the imaging parameters were as follows: beam energy: 17.5 keV and sample rotation degree: 180°; sample rotation speed was set to 0.4°/s, so the total scan time was approximately 8 minutes with about 900 projections. The exposure time of each projection was 6 ms. The distance between the sample and the CCD was 90 cm. The image pixel size was 9 *μ*m.

### 2.3. Data Processing

The stomach is a hollow digestive organ with rich blood vessel network. It is a challenging task for scientists to analyze them in 2D projection images because of the tissue overlap, image noise, small size of the vessels, and pathological abnormalities. In order to obtain the 3D microvessel information, volume reconstruction and necessary image processing were performed in this study.

#### 2.3.1. Feature Extraction

The images we got directly from PC-CT were projection images. A CT volume was reconstructed using filtered back projection (FPB) method [16]. Subsequently, blood vessels should be segmented. However, the microvessels, after injection of contrast agent, have different gray values compared to the larger vessels. We handle this problem by microvessel enhancement and thresholding segmentation.

The microvessels in the CT slice are the small spots of various gray level surrounded by soft-tissue area. It is impossible to segment them by one simple threshold. Therefore, a gray-scale reconstruction algorithm was used for the microvessel enhancement [17]. Once the microvessels were enhanced, Otsu thresholding method was applied to the CT volume to segment the blood vessels [18]. For the 3D visualization of the vessels, surface rendering based on marching cubes algorithm was applied, which allowed a clear visualization of the complex microvascular systems.

#### 2.3.2. MVD Calculation

MVD has been used as a parameter to quantify the vessel angiogenesis. A general approach for MVD quantification is to count vessels within a highly vascularized region on a 2D histological slice. However, there is still no standard for MVD quantification, and results can be conflicting as this kind of MVD is restricted in 2D, which cannot give a comprehensive description of the tumor angiogenesis. In this study, we use the feature of vessel centerline to define a 3D MVD.

The centerline is an important feature of the vessel. It can be used to monitor the vessel growth, distribution, deformation, and furthermore the progression of diseases. Therefore, in this study, we used vessel centerline to calculate a 3D MVD. To do so, we used parallel thinning algorithm to find the 3D centerline [19]. Again, it is a morphological operation that converts selected foreground pixels to 1-pixel-wide lines by iteratively removing pixels inside the shape to shrink it without shortening it or breaking it apart. Because parallel thinning algorithm is very sensitive to the surface of the vessels, pruning was carried out after thinning the vessel centerline.

Then, the 3D MVD can be defined as follows:

(1) MVD=∑voxels on the blood vessel centerline∑voxels in the region of interest.

Here, we use the blood vessel centerline as the feature to describe the vessel network. The ratio between the number of voxels in these centerlines and the number of all the voxels in the volume of interest can reflect the density of vessels in this region, which is the 3D MVD.

## 3. Results

**Figure 2:**
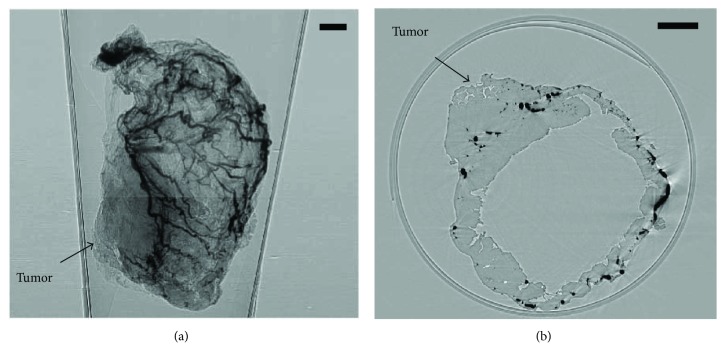
The projection and CT reconstruction images of PC-CT. (a) One projection image; (b) one CT slice (bar: 1 mm).

The imaging data contained both normal stomachs and malignant ones. For each sample, nearly 900 projection images were taken during the PC-CT experiment (Figure 2(a)). The CT slices were reconstructed by FBP algorithm (Figure 2(b)).

**Figure 3:**
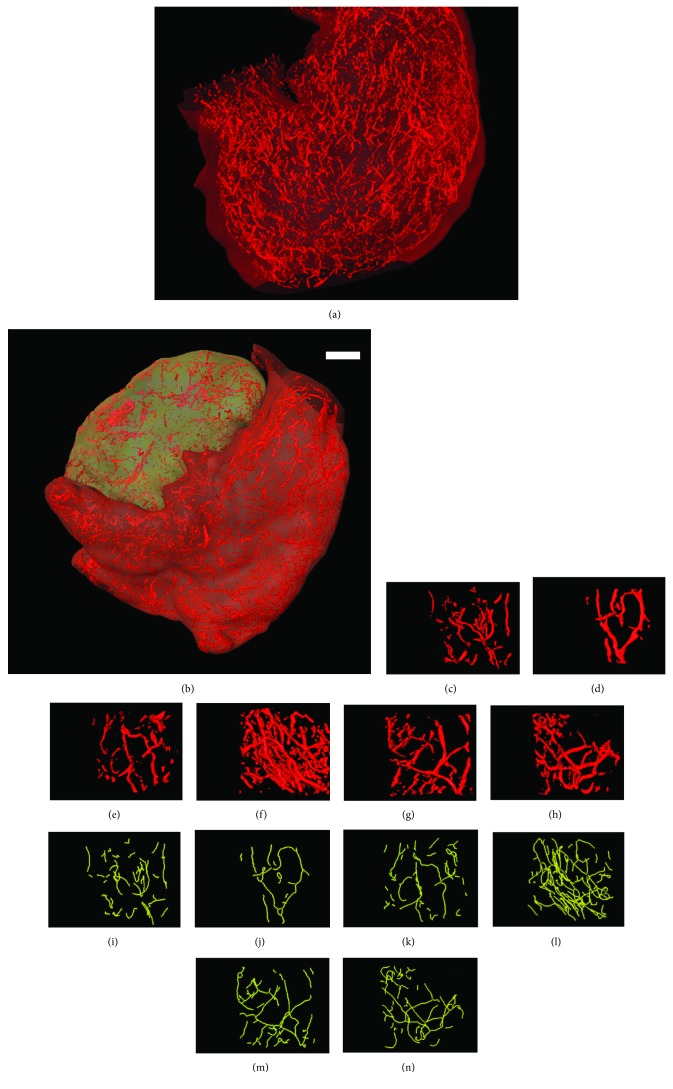
3D model of a normal stomach and one with a tumor. (a) 3D model of a normal stomach; (b) 3D model of a stomach with cancer; (c–h) volume of interest (VOI) in models A and B; (i–n) centerline extraction results of (c–h). Bar: 1 mm.

**Figure 4:**
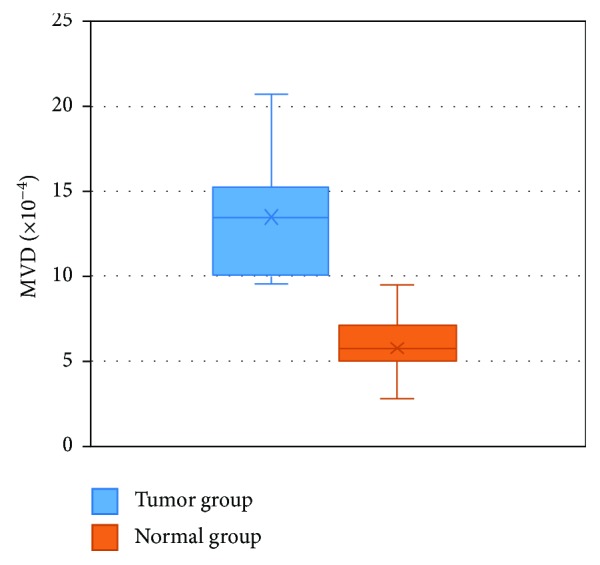
MVD statistics of the two sample groups.

The physiological function of the stomach determines its shape like a bag whose surface has a series of folds. Normal blood vessels grown in the stomach are elastic and have uniform diameter, smooth wall, normal distribution, and regular bifurcations.  Figure 3(a) showed the 3D model of the vessel network of a normal stomach, noted as model A. As to the cancer samples, there were a large number of blood vessels accumulated to the tumor with abnormal architecture including rigidity, rough wall, and chaotic distributions, as shown in Figure 3(b), noted as model B. We randomly selected three volumes of interest (VOIs) in models A and B, respectively, and gave an enlarged view of them in Figures 3(c)–3(h). These VOIs have the same size of 100 × 100 × 100 pixels. In order to give a quantitative evaluation of the samples, MVD was calculated. We showed the centerline extraction results in Figures 3(i)–3(n), and then the MVDs were 7.17 × 10^−4^, 3.99 × 10^−4^, 7.07 × 10^−4^, 20.72 × 10^−4^, 10.01 × 10^−4^, and 9.72 × 10^−4^, corresponding to VOIs C to H. The MVD in the tumor group was significantly higher than that in the normal group. Then, we selected ten VOIs in each of the samples and obtained the statistical distributions of the MVD in Figure 4. In the normal group, the MVD changed from 2.87 × 10^−4^ to 9.52 × 10^−4^, and the average was 5.97 × 10^−4^, whereas the MVD in the tumor group changed from 9.53 × 10^−4^ to 20.72 × 10^−4^ with an average of 13.38 × 10^−4^. Evident difference had been found by Student's *t* testing (*p* < 0.05).

**Figure 5:**
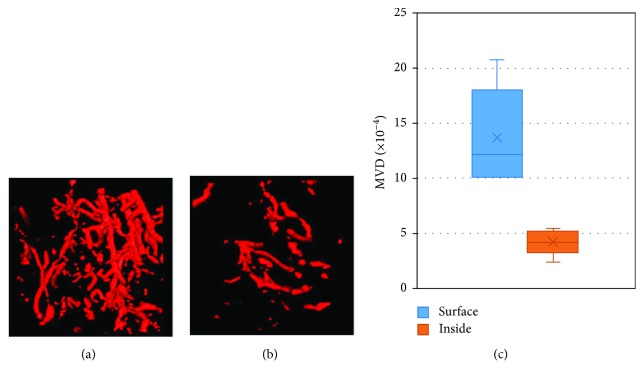
MVD comparison between the tumor surface and inside. (a) One VOI of tumor surface; (b) one VOI of tumor inside; (c) MVD of the two groups (*n* = 10 per group).

To investigate the vessel distribution of stomach tumor, we randomly chose 10 VOIs in the outer layer and 10 VOIs inside the tumor to examine the MVD. As shown in Figure 5, the MVD on the tumor surface was much higher than that on the one inside it (*p* < 0.01), reflecting that the tumor surface has higher vessel angiogenetic activities.

**Figure 6:**
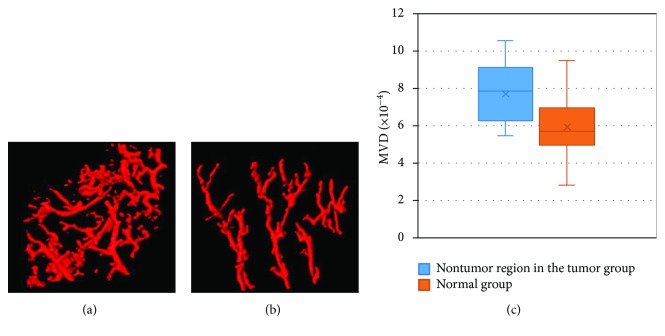
MVD comparison between nontumor region in the tumor group and the normal group. (a) One VOI of nontumor region in the tumor group; (b) one VOI of the normal group; (c) MVD statistics of the two groups (*n* = 10 per group).

To determine the effect of tumor growth to the nontumor region, we tested the MVD of the nontumor region in the tumor group (10 VOIs) and compared them with the normal group (10 VOIs). Interestingly, even in the nontumor region, tumor growth still had strong impact on the vessel system (Figure 6). The MVD had statistically significant difference (*p* < 0.05).

According to the 2D MVD method, if we choose one slice in the 3D VOI and count the vessel numbers in the image, we can get 2D MVD. Following this approach, we got the 2D and 3D MVDs in Table 1. The 2D slices were manually selected to the tenth and fiftieth slices of each VOI. It stands to reason that the tumor vessel distribution was heterogeneous. The viewing angles of the 2D slice largely affect the result of 2D MVD. For example, the 2D MVD of volumes 4, 5, and 6 changed dramatically from the tenth slice to the fiftieth. Therefore, a specific slice may not well reflect the tumor angiogenesis. A 3D MVD from a volume of microvessels can assure much robust and consistent outcome.

**Figure 7:**
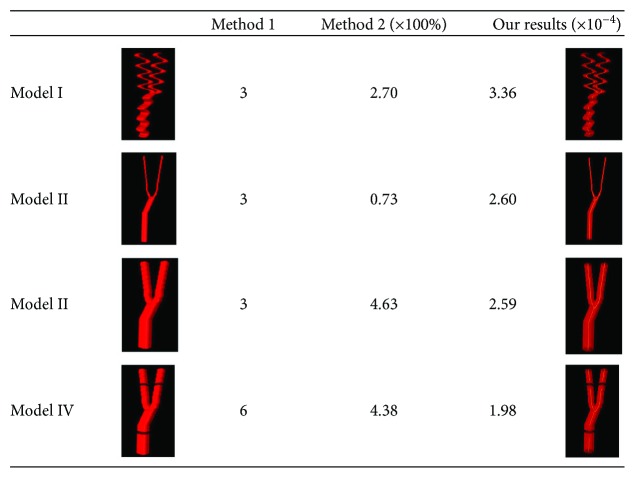
The 3D MVD parameters from three methods.

In light of 3D MVD, we also tried two other methods in four vessel model cases: (1) counting the vessel numbers in the VOI and (2) counting the vessel voxels in the VOI. Case I was a vessel tree with big curvature; cases II and III were two vessel trees with different diameters; and case IV was the vessel tree with three discontinuities representing some detection misses. The results were shown in Figure 7. No matter how the shape changes or the diameter changes, the method (1) always gave the same results, which means it cannot be used as a valid method. As for the method (2), the results were greatly impacted by the blood vessel diameter. Both of the methods cannot provide reasonable descriptions of the vasculature distributions compared to the proposed method.

## 4. Discussion

Accumulating evidence has shown that angiogenesis has close relationship with tumor occurrences and metastasis and is one of the major indicators for malignancy [20, 21]. According to recent studies, CSC might be closely involved in the angiogenetic process [22], which shed a new light on tumor treatment, although there are still uncertainties in the process [23]. Our study presents a new way for gastric cancer microvascular observation in 3D. It provides a comprehensive view and quantitative description for the tumor vascularization and may help understanding the CSC factor in cancer angiogenesis.

The vessels in the tumor group can be differentiated from the normal group in great detail, such as the curvature, vessel wall, bifurcations, and so on. Moreover, as showed in our observations, the microvessels were crowded on the tumor surfaces. Even in the nontumor area in the malignant samples, the 3D MVD was higher than that in the normal group, which meant the microvascular distribution was still significantly denser than the normal ones.

It is proved that MVD is an indicator of tumor angiogenesis, which has close relationship with tumor growth and metastases. Moreover, it can reveal the malignancy of the gastric cancer and serve as one of the predictors of prognosis [24]. Considering the quantitative description of the microvessel density, there is still no gold standard. This directly leads to the discrepancies in cancer angiogenesis study. Weidner's two-dimensional MVD has been an accepted measure in the field. However, it may miss the “hotspot” or may not present a complete view of the tumor as demonstrated by our experiment in Table 1. 3D MVD, in contrast, is more robust, and the evaluation of microvessels is more direct and comprehensive.

For microvessel imaging, microscopy is the most common method used in recent studies. Either confocal or fluorescence microscopy can provide high resolution of the blood vessels and even cells [25]. However, as the improvement of the precision, the field of view decreased sharply, and the imaging depth is limited. The specimens should be specially preserved. It is very complicated, time-consuming, and resource-demanding work to get the 3D images of the gastric cancer by continuous histological slice and image processing. By CT reconstruction and image segmentation, our phase-contrast imaging approach has high imaging resolution and soft-tissue contrast; moreover, the field of view is suitable for normal biological samples.

A limitation in our study is the data harvesting. During CT scans, the stomach samples sometimes atrophied because the stomach wall was too thin to resist deformation, which made a big trouble for our subsequent CT reconstruction. The microvessels in the image were only a few pixels in width. Vessel deformation was very difficult to be calibrated and finally caused data loss.

## 5. Conclusions

Our work demonstrated that phase-contrast imaging can produce clear view of the gastric cancer and its vascular systems, which have been confirmed by histological sections. We can qualitatively understand the microvessel distribution and detect pathological changes of the vessel morphology. After vessel image segmentation and centerline extraction, our results showed that 3D MVD is closely correlated with the pathological changes of gastric cancer. The proposed 3D MVD can provide reliable quantitative measure of the angiogenesis in gastric cancer. The new metric can better facilitate the evaluation of the cancer progress and prognosis than the 2D measure.

## Figures and Tables

**Table 1:** MVD of ten VOIs.

	VOI number	3D MVD × 10^−4^ (voxels of vessel centerline per 100 × 100 × 100 voxels)	2D MVD slice number 10 (vessel number per 100 × 100 pixels)	2D MVD slice number 50 (vessel number per 100 × 100 pixels)
Tumor group	1	15.18	16	20
	2	10.07	9	7
	3	20.72	24	33
	4	12.04	11	21
	5	10.51	10	17

Normal group	6	4.22	19	4
	7	4.1	7	6
	8	6.45	5	11
	9	6.61	10	10
	10	7.9	13	8
